# Bryophyte Species Richness on Retention Aspens Recovers in Time but Community Structure Does Not

**DOI:** 10.1371/journal.pone.0093786

**Published:** 2014-04-07

**Authors:** Anna Oldén, Otso Ovaskainen, Janne S. Kotiaho, Sanna Laaka-Lindberg, Panu Halme

**Affiliations:** 1 Department of Biological and Environmental Science, University of Jyväskylä, Jyväskylä, Finland; 2 Metapopulation Research Group, Department of Biosciences, University of Helsinki, Helsinki, Finland; 3 Finnish Natural History Museum LUOMUS, Botany unit, University of Helsinki, Helsinki, Finland; Institute of Botany, Chinese Academy of Sciences, China

## Abstract

Green-tree retention is a forest management method in which some living trees are left on a logged area. The aim is to offer ‘lifeboats’ to support species immediately after logging and to provide microhabitats during and after forest re-establishment. Several studies have shown immediate decline in bryophyte diversity after retention logging and thus questioned the effectiveness of this method, but longer term studies are lacking. Here we studied the epiphytic bryophytes on European aspen (*Populus tremula* L.) retention trees along a 30-year chronosequence. We compared the bryophyte flora of 102 ‘retention aspens’ on 14 differently aged retention sites with 102 ‘conservation aspens’ on 14 differently aged conservation sites. We used a Bayesian community-level modelling approach to estimate the changes in bryophyte species richness, abundance (area covered) and community structure during 30 years after logging. Using the fitted model, we estimated that two years after logging both species richness and abundance of bryophytes declined, but during the following 20–30 years both recovered to the level of conservation aspens. However, logging-induced changes in bryophyte community structure did not fully recover over the same time period. Liverwort species showed some or low potential to benefit from lifeboating and high potential to re-colonise as time since logging increases. Most moss species responded similarly, but two cushion-forming mosses benefited from the logging disturbance while several weft- or mat-forming mosses declined and did not re-colonise in 20–30 years. We conclude that retention trees do not function as equally effective lifeboats for all bryophyte species but are successful in providing suitable habitats for many species in the long-term. To be most effective, retention cuts should be located adjacent to conservation sites, which may function as sources of re-colonisation and support the populations of species that require old-growth forests.

## Introduction

Despite their great importance for biodiversity conservation, ecosystem services and climatic control, only 12.5% of the world's forests are under legal protection while the rest are being exploited or converted for multiple purposes [1]. For the conservation of biodiversity 12.5% is not enough, and therefore it is essential that sustainable forestry practises are developed in managed forests [2]–[4]. To preserve biodiversity while still retaining the economic viability of forestry, a major opportunity is to use silvicultural approaches that mimic natural disturbances [5]–[8].

Retention forestry is an approach where some structures and organisms of the forest are intentionally retained during logging actions, mimicking the biological legacies left by natural disturbances [6], [7], [9]–[11]. It is applied widely in boreal and temperate forests for biological, ecological and social reasons [10]. Three main ecological objectives of retention forestry are: 1) *‘lifeboating’ species and processes over the regeneration of the forest*, 2) *enriching re-established forest stands with structural features*, and 3) *enhancing the connectivity of the landscape* [6]. Lifeboating species over the regeneration phase implies that due to retention the species can occupy the stand continuously over time [12]. In contrast, structural enrichment refers to the presence of specific microhabitats that can be inhabited by such forest species that were eliminated after logging but are able to re-colonize the structures after the surrounding forest has re-established [6]. Structural enrichment can also be relevant for disturbance-phase species that colonise the stand after logging [12]. Finally, tree retention enhances landscape connectivity if individuals can disperse through the stand due to retention [12]. Thus, lifeboating and structural enrichment function locally while landscape connectivity functions at larger spatial scales. Lifeboating, landscape connectivity and structural enrichment for disturbance-phase species are temporally relevant immediately after logging and continuously during the lifespan of the retained structures although their importance decreases as the surrounding forest re-establishes. In contrast, the importance of structural enrichment for forest species increases during the lifespan of the structures.

Green-tree retention refers specifically to leaving some living trees on a logged stand. The majority of studies have concluded that compared to clear-cutting, green-tree retention improves at least the short-term survival of several taxa and thus appears to be effective in promoting lifeboating [12]. However, the success of green-tree retention in promoting lifeboating varies between taxa; notably bryophytes (mosses and liverworts) survive poorly after logging [12]–[15]. Most bryophyte species can tolerate periods of desiccation, but species of mesic habitats are damaged by rapid drying or severe desiccation [16]. Their survival may be decreased after logging-induced changes in humidity and light conditions [17], [18].

Epiphytes are expected to depend strongly on lifeboating because retention trees provide them with substratum that is missing on clear-cuts. However, microclimatic changes may cause their decline on retention trees compared to similar trees left in unlogged forests [12], [17]. Lõhmus et al. [17] found that two years after logging solitary retention trees had significantly less bryophyte species, lower bryophyte cover (%) and lower bryophyte vitality than trees in intact forests. Different species of bryophytes may show different responses to the changed microclimate depending on their life-form [17]: mat-, weft- or fan-forming species favour shady and/or moist conditions, while species that form small cushions are more common in more sunny and/or dry places [19].

It is possible that bryophytes are able to re-colonise young stands sooner if there are suitable substrata and source populations nearby, and therefore the structural enrichment of re-established stands may be more important for bryophytes than the short-term lifeboating [12], [20]. However, the long-term value of GTR for epiphytes has been insufficiently studied [12], [20]. Many epiphytic bryophytes commonly produce spores or asexual gemmae, which may facilitate the dispersal of the species between the patchily-occurring substrate trees [21]. This adaptation could aid the colonisation of the retention trees in the re-established stands.

European aspen (*Populus tremula* L.) supports specific and diverse epiphyte communities [17], [22]. The number of aspens in northern Europe has declined especially in protected areas due to e.g. the lack of large-scale disturbances and the browsing of saplings by herbivores [23]. If the decline of aspen continues as predicted, it is expected to result in regional extinctions of many aspen-associated species [23], [24]. Therefore aspen is considered to be a valuable species for retention [17], [25].

Here we investigate the value of retained aspen trees for epiphytic bryophytes in both the short-term lifeboating and the long-term potential of re-colonisation. We study retention aspens along a chronosequence of differently-aged retention sites to estimate the changes in bryophyte communities during 30 years after logging. We also compare the bryophyte communities of retention aspens with aspens in conservation areas (later ‘conservation aspens’), because there is a need to evaluate the retention approach relative to alternative conservation strategies such as setting aside permanent conservation areas [7]. We address three specific questions: 1) To what extent do bryophytes occupy retention aspens after logging, i.e. are retention aspens promoting lifeboating of bryophytes? 2) To what extent are bryophytes able to re-colonize retention aspens after a stand has re-established, i.e. are retention aspens functioning as structural enrichment for bryophytes? 3) Can retention aspens substitute conservation aspens in terms of maintaining biodiversity and ensuring the long-term persistence of populations? To address these questions, we build a hierarchical Bayesian model that utilises both species-level and community-level information in the data, and we use the parameterised model to ask how bryophyte species richness, abundance and community structure may change on an aspen after either retention or conservation.

## Material and Methods

### Study sites

**Figure 1:**
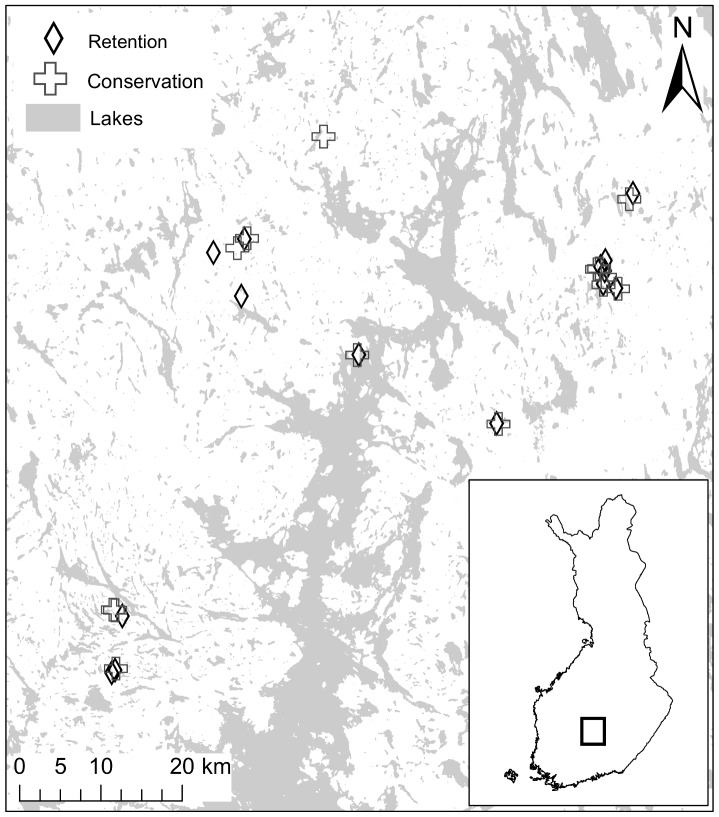
Map of study sites in Central Finland. © National land survey of Finland 2013.

The study sites were located in the southern boreal vegetation zone (see [26]) in Central Finland (61°53′N 25°42′E, Fig. 1) where the mean air temperature is 16°C in July and −8.5°C in January (average from 1971–2000) and the average precipitation is 600–650 mm year^−1^. The study was conducted in 2008 (between July and October) on 14 retention sites and 14 conservation sites that are state-owned and managed by Metsähallitus (Finnish forest and park service). Study permits were provided by Metsähallitus. The retention sites had varying times since logging (mostly between 2 and 12 years, but on three sites approximately 16, 27 and 30 years) and the conservation forests had varying stand ages (between 85 and 175 years, estimates derived from the database of Metsähallitus). The data was collected once and in the analyses we use the chronosequences formed by the retention sites with different times since logging and the conservation sites with different stand ages to reveal the effects of time. The conservation forests include strictly protected areas such as national parks and nature reserves as well as managed areas that have been set aside from management practices or are managed with very low intensity. Almost all of the conservation sites can be described as semi-natural, i.e. some signs of human actions can be found. Most of them have been used for intensive forestry prior to setting them aside for conservation.

The sites were typical boreal heath forests for the area, representing *Myrtillus* or *Oxalis*-*Myrtillus* type (see [27]). The dominant tree species was Norway spruce (*Picea abies* [L.] Karst) before the logging on the retention sites as well as at the time of the study on the conservation sites. The areas of the retention sites varied between 1 and 20 hectares, and the areas of the conservation forests varied between 3 and 75 hectares (estimates derived from the database of Metsähallitus). The locations, stand ages, areas and forest types of the study sites are presented in File S1.

### Data collection

At each retention site, we aimed to sample two aspens in each of seven size classes (diameter at breast height [130 cm]: 10-<20 cm, 20-<30 cm,…, 70–80 cm), but the total number of studied aspens varied from six to ten per site as a result of variation in available aspen size classes. The aspens within a size class were chosen randomly, including both solitary and grouped trees. For each retention site, the same number of aspens in each size class was studied at the nearest possible conservation site. Aspens were included in the study only if they were living, healthy and vertical (not leaning) to reduce the effect of such rare trees that are often exceptionally species-rich. In addition, to reduce the impacts of positive edge effects on retention aspens (see [28]) and negative edge effects on conservation aspens (see [29]), we included only aspens that were located at least 10 metres from forest edge in the retention sites or at least 30 metres from forest edge in the conservation sites. We studied a total of 102 retention aspens and 102 conservation aspens.

On each study aspen, all bryophyte species growing on the lowest two metres of the trunk were recorded. Only bryophytes growing directly on the bark or on other epiphytes were included, thus excluding those that grew on detritus or humus. If tree roots were exposed, then also those bryophytes that grew on the roots with a maximum distance of 20 cm from the trunk were included. The abundance of each bryophyte species on a trunk was measured as area covered (cm^2^). If the species covered a small area (a few cm^2^) the abundance was estimated. If the area covered was larger the maximal colony diagonals (d1 and d2 perpendicular to each other) were measured with a tape measure, creating a kite-shaped area, and the cover (%) of the species within the area was estimated and the abundance was then calculated as d1*d2*½*cover. Specimens were taken for microscopic identification whenever identifications were not possible in the field. The original data is available in File S1. One reference specimen of each observed species and all specimens of red-listed species have been deposited in the Natural History Collection of Jyväskylä University Museum (JYV). The nomenclature follows Ulvinen & Syrjänen [30] and the classification of red-listed species follows Syrjänen et al. [31]. Mosses and liverworts were analysed together.

### Statistical analysis

We analysed the data using the hierarchical community approach of Ovaskainen & Soininen [32]. The modelling approach enables us to discern the species-specific responses to environmental covariates, as well as to combine these species-specific responses into a community-level model. The combination of the species-specific models with the community-level model improves the parameterisation of especially rare species as it allows for borrowing strength from the other species [32]. In addition, the community model provides a parameter-sparse description of the entire community, which enables a simple analysis on how e.g. environmental dissimilarity translates into community dissimilarity. Here we present the main outlines of the modelling; detailed information on the mathematical formulation of the model and its statistical parameterization are provided in File S2.

For each species in our dataset, we built two separate models which share the same structure, but in one model the response variable is the presence-absence of a species on a tree, whereas in the other it is the abundance of a species on a tree conditional on the species was present on the tree. In the presence-absence model we applied logistic regression to model the probability that the species (*i*) is present on a tree (*j*) on a site (*k*), 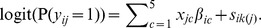



The linear predictor includes the values of five environmental covariates on the tree (*x_jc_*) multiplied by their effects on the species (*β_ic_*) plus a site-level random effect on the species (*s_ik(j)_*). We included five covariates (*c*):

1. intercept (modelling the rarity of the species)
2. diameter of the tree (log-transformed)
3. site type, i.e. an indicator variable separating retention aspens (*x_j3_* = −1) from conservation aspens (*x_j3_*  = 1)
4. time since logging (unit year, relevant only for retention aspens)
5. stand age (unit year, relevant only for conservation aspens).

The covariates 2, 3, 4 and 5 were normalised to zero mean and unit variance to make their effect sizes comparable with each other. For each covariate and for each species, we estimated a regression coefficient that measures the influence of the covariate on the species (i.e. the response of the species to the covariate, *β_ic_*).

The site-level random effect (*s_ik(j)_*) models the response of the species to such variation among sites that is not captured by the site-level covariates (3, 4 and 5). The site-level random effects were assumed to be distributed according to the multivariate normal distribution which involves two components: environmental variation that is shared among the species and environmental variation to which the species respond to independently.

In the abundance model we applied linear regression for log-transformed data to model the abundance (unit cm^2^) of the species (*i*) on a tree (*j*) on a site (*k*),
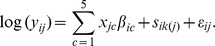



The abundance model was built similarly to the presence-absence model except that a normally distributed residual (*ε_ij_*) was also included. The residual was not included in the presence-absence model as it is not identifiable in a logistic regression.

For both the presence-absence model and the abundance model the species-specific models were combined into a model of the entire species community by assuming that the regression coefficients of the species (*β_ic_*) are distributed multinormally as 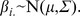



Here ***β_i._*** is a vector that is formed by the responses (regression coefficients *β_ic_*) of the species *i* to the five covariates. ***μ*** is a vector that is formed by the average responses (*μ_c_*) of the species to the five covariates. ***Σ*** is a variance-covariance matrix that includes variation among species in their responses to the environmental covariates (variances on the diagonal elements) and co-variation among responses to different covariates (covariances on the off-diagonal elements),

We fitted the presence-absence model and the abundance model independently of each other. We used Bayesian inference, and thus prior distributions needed to be defined for the community-level parameters ***μ*** and ***Σ***, for the parameters related to the site-level random effects, and for the residual variance parameter (relevant only for the abundance model). As detailed in File S2, we used as uninformative priors for all model parameters as was technically possible and we fitted the models to data using a slightly adapted version of the MCMC scheme of Ovaskainen & Soininen [32]. The estimation was performed with Mathematica 7.0. The resulting estimates of the species-specific regression coefficients *β_ic_* are available in File S3.

### Scenario comparisons

We used the fitted models to compare the development of bryophyte communities between retention and conservation aspens. We considered as the starting point an aspen tree with 30 cm diameter located in a forest with a stand age of 80 years. We then assumed that the forest was logged (in which case the aspen became a retention tree) or conserved, and examined how the community on the aspen would evolve over time until 30 years since logging (for the retention aspen), or until the stand age reached 150 years (for the conservation aspen). We assumed that the diameter of the aspen grew linearly so that it reached 60 cm for the stand age of 150 years. For these scenarios, we predicted the expected species richness (based on the presence-absence model) and the abundance (dm^2^) of all bryophytes (based on probability of presence from the presence-absence model multiplied by abundance conditional on presence from the abundance model). We also predicted how similar the community structure (predicted by the presence-absence model) would be to a reference community (R) of an old-growth aspen, defined here as the modelled community of an aspen that has 60 cm diameter and occurs on a conservation site with stand age of 150 years. We followed Ovaskainen & Soininen [32] in measuring community similarity between reference (R) and focal (F) sites. Community similarity was calculated through the similarity of environmental covariates (vectors **x** *_R_* and **x** *_F_*) weighted by the importance of the covariates to variation in species responses (measured by the matrix ***Σ***). Details are given in File S2.

### Bryophyte reaction groups

We used the median estimates of the regression coefficients from the presence-absence model to classify the species to four reaction groups: ‘disturbance-favouring’, ‘lifeboated’, ‘re-colonising’ and ‘old-growth-favouring’. Species were defined as disturbance-favouring if their occurrence was higher on retention aspens than on conservation aspens (*β_i3_* <−0.4), i.e. they benefited from the logging disturbance. Species that simultaneously did not show strong preference for retention or conservation aspens (−0.4< *β_i3_* <0.4) and did not increase or decrease with time since logging (−0.4< *β_i4_* <0.4) were considered as species that were successfully lifeboated on the retention aspens. Of the species that were not lifeboated successfully, we discerned re-colonising species as those whose occurrence increased strongly with time since logging (*β_i4_* >0.4). Old-growth-favouring species showed strong preference for conservation aspens over retention aspens (*β_i3_* >0.4) and simultaneously did not show strong re-colonisation over time on retention aspens (*β_i4_* <0.4). We note that the limits (*β_ic_*  = ±0.4) are arbitrary and thus the classification of species with reactions close to the limits is uncertain.

All bryophyte species were classified to the reaction groups, but we note that the potential value of aspen retention trees is highest for those species that are most dependent on aspens as their substrate. Therefore we focus in particular on species that are obligately or primarily epiphytic rather than species that are only occasionally epiphytic. The species were classified to the three groups based on their ecology in Finland [33] and the following criteria: obligately epiphytic species grow almost exclusively on deciduous tree trunks (usually on aspen) and very rarely on other substrates, primarily epiphytic species grow most often on deciduous tree trunks but also on other substrates, whereas occasionally epiphytic species grow sometimes on deciduous tree trunks but are common on other substrates as well.

## Results

### Bryophyte occurrence and abundance

**Figure 2:**
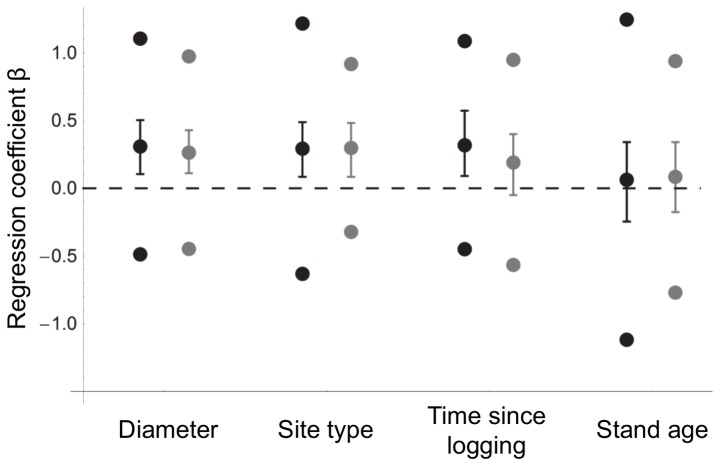
The responses (standardised regression coefficient) of bryophytes to the four covariates included in the study. Black symbols correspond to the presence-absence model and grey symbols to the abundance (conditional on presence) model. The middle points and bars show the average responses of the species to each of the covariates (posterior mean and 95% central credibility interval for the vector ***μ***). The lower and upper points indicate the range of responses shown by 95% of the species (posterior means for ***μ ±*** 2SD, where the SD are the standard deviations obtained from the diagonal elements of the matrix ***Σ***). Diameter shows the effect of increasing aspen size and site type separates retention aspens (−1) from conservation aspens (+1). Time since logging is relevant only for retention aspens whereas stand age is relevant only for conservation aspens.

Altogether 46 moss and 14 liverwort species were found on the study aspens (see File S3 for species list). The occurrence and abundance of bryophytes were greater on conservation aspens than on retention aspens (Fig. 2, site type: the 95% highest posterior distribution of *μ*_3_ is positive in both models). Further, both the occurrence and the abundance of bryophytes increased with increasing aspen size (Fig. 2, diameter). On the retention aspens the occurrence and to some extent also the abundance increased with increasing time since logging, whereas on the conservation aspens stand age had no overall effect on occurrence or abundance (Fig. 2, time since logging and stand age). Bryophyte species showed considerable variation in their responses to all of these environmental covariates ranging from negative to positive responses (Fig. 2).

**Figure 3:**
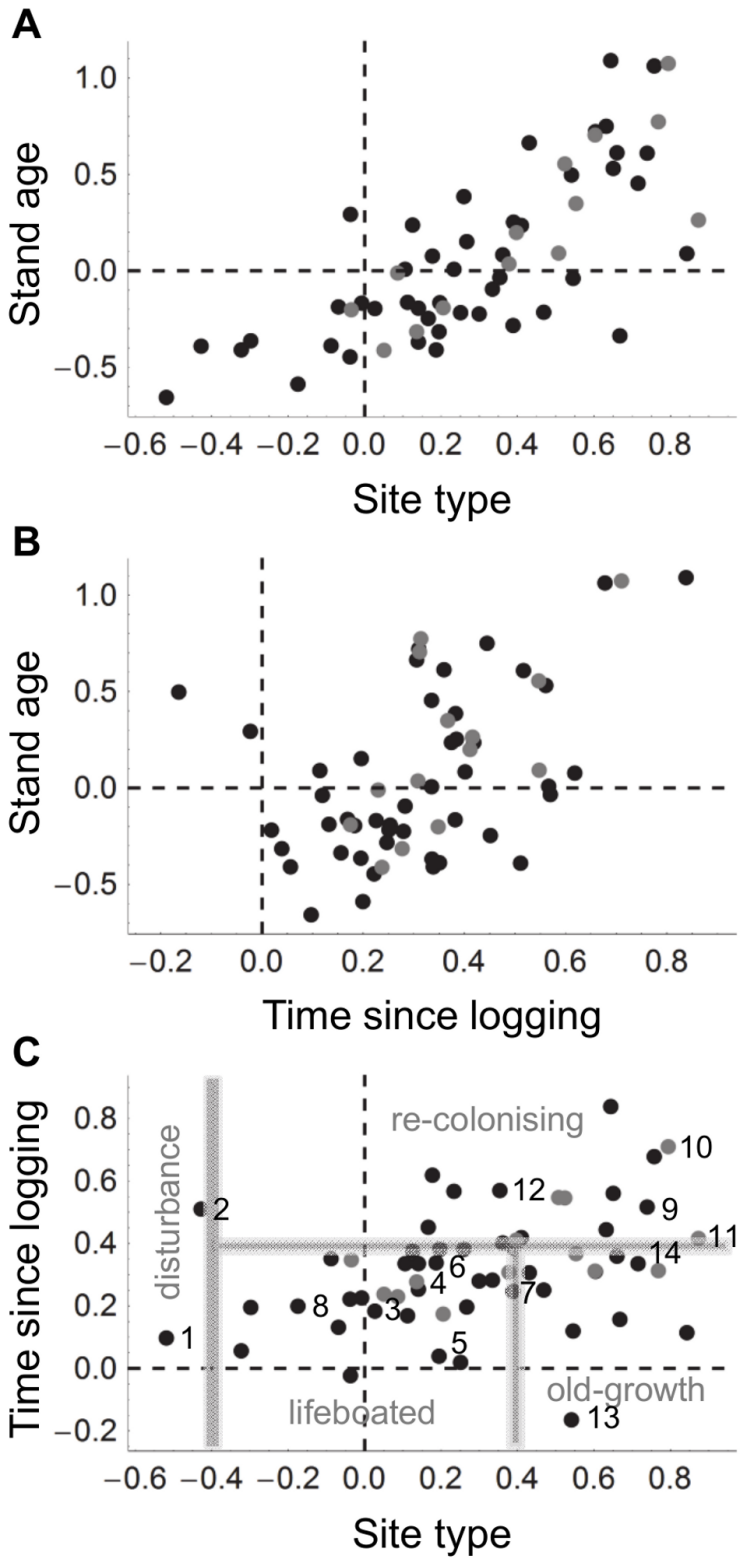
Co-variation among species specific regression coefficients between the covariates. a) Site type and stand age, b) time since logging and stand age, and c) site type and time since logging. Negative values of site type refer to retention sites and positive to conservation sites. Mosses are depicted in black and liverworts in grey. Species-specific regression coefficients are based on the presence-absence model. See File S3 for the species specific regression coefficients for each covariate and Table 2 for the numbered species in c).

The estimate of the variance-covariance matrix of responses of different species to the covariates reveals a number of correlations (Table 1). Particularly, species that were more likely to occur on conservation sites with high stand age were also more likely to occur on conservation aspens than on retention aspens (Fig. 3a). When such species occurred on retention aspens, they were more likely to occur on retention sites with long time since logging (Fig. 3b, Table 1).

**Table 1:** Correlations among the species-specific responses to the environmental covariates.

	intercept	diameter	site type	time since logging	stand age
intercept		−0.08 (0.65)	0.01 (0.50)	0.003 (0.45)	0.04 (0.41)
diameter	0.11 (0.29)		−0.04 (0.58)	−0.29 (0.92*)	−0.32 (0.95*)
site type	0.19 (0.20)	0.002 (0.49)		0.22 (0.18)	0.51 (0.002*)
time s. logging	0.38 (0.03*)	0.005 (0.51)	0.10 (0.34)		0.27 (0.10*)
stand age	−0.06 (0.61)	−0.29 (0.91*)	0.15 (0.26)	0.06 (0.41)	

Correlations from the presence-absence model are given above the diagonal and correlations from the abundance model are below the diagonal. The correlation coefficient is the posterior mean estimate and the value in parenthesis the posterior probability by which the correlation is negative. Cases for which the correlation was positive or negative with at least 90% posterior probability are indicated with an asterisk (*). For more details see methods.

### Scenario comparisons

**Figure 4:**
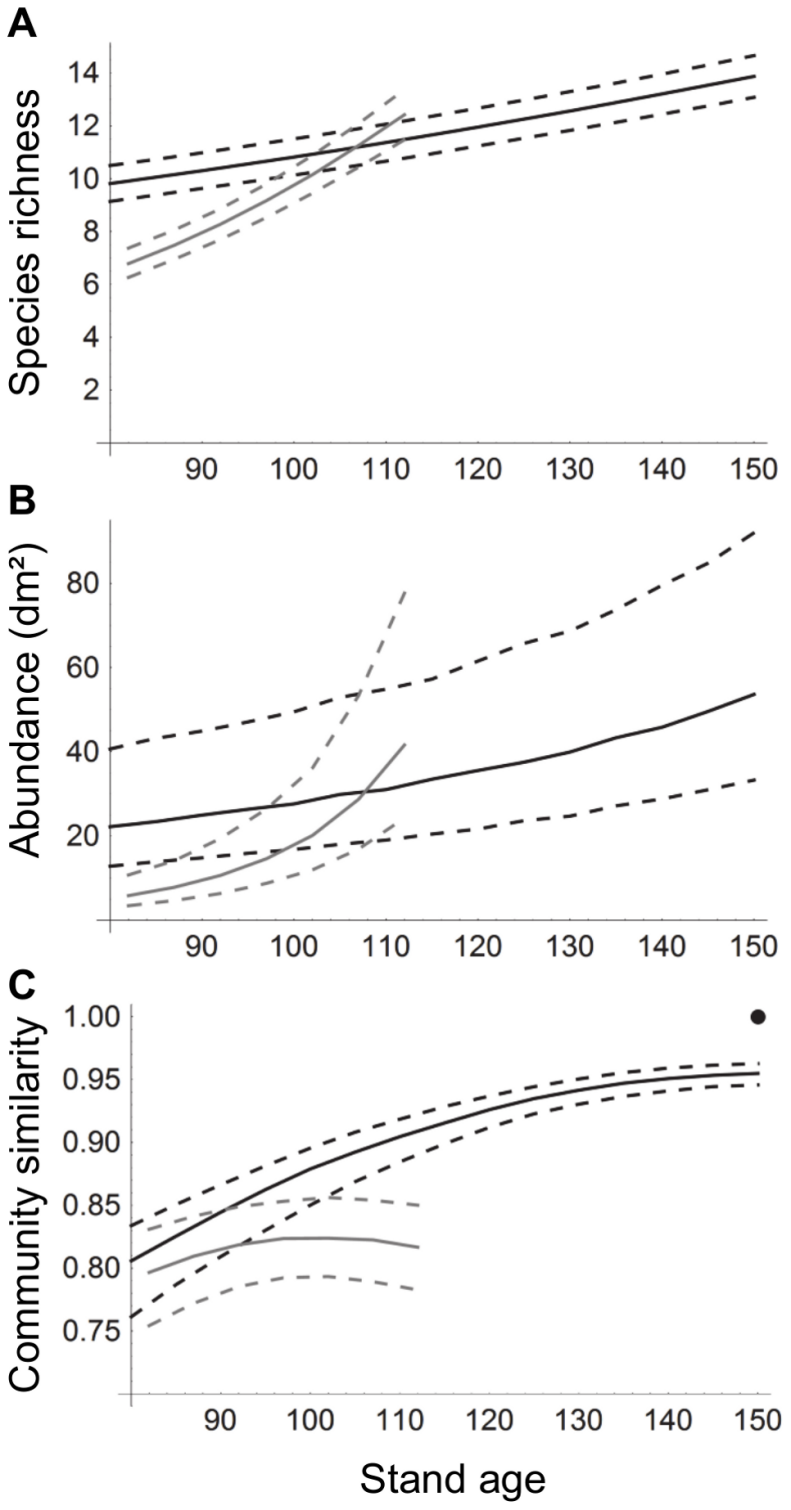
Relationship between stand age and bryophyte species richness (a), abundance (b) and community structure (c). The black lines correspond to aspens in uncut conservation sites, the grey lines correspond to retention aspens in forests that are cut at the stand age of 80 years. Continuous lines show median estimates, dashed lines the interquartile range. Community similarity (c) is measured against a modelled reference community (marked with •) of an aspen that has 60 cm diameter and that occurs in an uncut forest with stand age 150 years. Community structure is based on the presence-absence model.

Using the fitted model, we estimated the changes in the species richness and abundance of bryophytes on retention and conservation aspens over time (Fig. 4). On conservation aspens both species richness and abundance increased, while on retention aspens the species richness and abundance declined initially, but after 20–30 years recovered to the level of the conservation aspens (Figs 4a and 4b).

The community similarity of both retention and conservation aspens was compared to the modelled reference community. As expected, the community on the conservation aspens evolved steadily to be more similar to the reference community (Fig 4c). Note that the correlation does not reach the value of 1 even when the environment of the focal community is identical to that of the reference community because of random variation among sites. On retention aspens, logging changed the community structure so that soon after logging the bryophyte community on retention aspens is less similar to the reference community than it was before the logging. As time since logging increased, community similarity to the reference community increased but with diminishing increments. Thus, soon after the logging, the estimated trajectory of the development of community similarity on the retention aspens deviated from that of the conservation aspens (Fig 4c).

### Bryophyte reaction groups

All species were classified to four reaction groups depending on their responses to site type and time since logging (Fig. 3c). Two of the species were classified as disturbance-favouring, 30 species as lifeboated, 16 species as re-colonising and 12 species as old-growth-favouring (see File S3 for species list). Examples of species in each reaction group are provided in Table 2, including all of those obligately or primarily epiphytic species that occurred on at least four aspens in our data set as well as three occasionally epiphytic species that have extreme reaction values.

**Table 2:** Examples of species in the four reaction groups (disturbance-favouring, lifeboated, re-colonizing, old-growth favouring; the classification is based on the presence-absence model).

No.	Species	Status	Epiphyte	Group	Life-form
Disturbance-favouring					
1	*Orthotrichum obtusifolium*	LC	Obligate	Moss	Small cushion
2	*Pohlia nutans*	LC	Occasional	Moss	Small cushion
Lifeboated					
3	*Amblystegium serpens*	LC	Primary	Moss	Thread-like mat
4	*Campylophyllum sommerfeltii*	LC	Primary	Moss	Thread-like mat
5	*Orthotrichum gymnostomum*	VU	Obligate	Moss	Small cushion
6	*Orthotrichum speciosum*	LC	Obligate	Moss	Small cushion
7	*Pylaisia polyantha*	LC	Primary	Moss	Rough mat
8	*Sciuro-hypnum populeum*	LC	Primary	Moss	Rough mat
Re-colonizing					
9	*Dicranum montanum*	LC	Primary	Moss	Short turf
10	*Ptilidium pulcherrimum*	LC	Occasional	Liverwort	Thread-like mat
11	*Radula complanata*	LC	Primary	Liverwort	Smooth mat
12	*Sanionia uncinata*	LC	Primary	Moss	Rough mat
Old-growth-favouring					
13	*Hylocomium splendens*	LC	Occasional	Moss	Weft
14	*Neckera pennata*	VU	Obligate	Moss	Fan

The list includes all obligately or primarily epiphytic species with ≥4 observations and three occasionally epiphytic species. No. refers to the numbering of species in Fig. 3c.

## Discussion

### Are retention aspens promoting lifeboating?

Both species richness and abundance of bryophytes on retention aspens declined shortly after the surrounding trees were logged. These results support the earlier views that in the short-term the habitats provided by retention trees are poor for many bryophytes in contrast to the higher success for several other taxa [12], [14], [17], [34]. However, the conclusion about the functionality of the retention approach depends on whether we compare it to clear-cutting or conservation. In the case of epiphytic species, retention sites are obviously more valuable than clear-cut sites which do not provide any suitable substrate. In our study, an average retention tree (diameter 30 cm) was able to support on average seven bryophyte species immediately after logging (Fig 4a) and therefore each retention aspen functions as a lifeboat for several species. On the other hand, based on our estimates an average of three species and more than half of bryophyte abundance on each retention aspen are lost (Figs 4a and 4b).

When compared to humid and shady forests, logged areas have increased illumination level, temperature variation, wind velocity and evaporation level, and lower atmospheric humidity [35]–[37]. Bryophytes are known to be sensitive to such changes in microclimate [17], [37], whereas epiphytic lichens, i.e. the other major epiphytic group, can acclimate physiologically to changes in microclimatic conditions and perhaps even increase their survival after retention logging [17], [38]. Our findings confirm earlier studies concluding that microclimatic effects of logging on bryophytes are drastic during the first 2–3 years but after that the bryophyte community stabilises, i.e. there is less change during the following 3–8 years [39], [40]. We note that the retention level is comparatively low in Finland [10]. Notably higher retention levels might result in less drastic declines because higher amounts of surrounding retention trees would provide more protection from microclimatic changes and because a large amount of retention trees would probably result in a larger amount of microhabitats and therefore the trees could complement each other.

Based on our classification, the occurrence of 30 species (50% of all species) on retention aspens was more or less similar to those in conservation aspens. This result suggests that for these species retention aspens do indeed function as successful lifeboats. The successfully lifeboated species include both mosses and liverworts. Among them are four primarily epiphytic mosses that form mats: *Amblystegium serpens*, *Campylophyllum sommerfeltii*, *Pylaisia polyantha* and *Sciuro-hypnum populeum*. Mats survive poorly in very dry or sunny conditions, but they grow close to the substrate and therefore moisture retention may be efficient enough for growth in somewhat dry or light conditions [19]. Among the lifeboated species are also the two cushion-forming obligate epiphytes *Orthotrichum speciosum* and *O. gymnostomum*. *Orthotrichum gymnostomum* is a red-listed aspen specialist that prefers forests with a protective microclimate but occurs also at open sites [41], possibly even colonising retention trees [20]. The success of small cushions on the open retention sites is expected because the cushion form enables efficient water storage and light use [19].

On the other hand, 28 species (47% of all species) showed low potential to benefit from lifeboating as they were much more common on conservation aspens than on retention aspens. Some of these species were often present in mature forests (no response to stand age), and therefore they had the opportunity for lifeboating, but apparently they suffer from the changed conditions after logging. Changes in microclimate is the most likely explanation as dried shoots of several species were commonly observed on the aspens of 2–3 years previously logged sites, while mechanical damage from e.g. logging machinery, ice or herbivores was observed only rarely. The majority of the species for which retention aspens do not function as lifeboats occur primarily in old-growth forests (strong positive response to stand age) and for them old-growth conservation areas are needed to support viable populations. Among them is the red-listed *Neckera pennata*, which is a long-living fan-forming moss that in the boreal zone is most often found on large aspens in natural, moist spruce forests [42]. Its growth and survival respond negatively to edge effects [43] and in a recent transplantation experiment it showed decreased shoot lengths and vitality on retention trees [29].

### Are retention aspens functioning as structural enrichment?

We estimated that some 20–30 years after logging both species richness and abundance of bryophytes on retention aspens would recover to the level of those on conservation aspens. We had in our chronosequence data set only three sites that had been logged more than 15 years earlier, and therefore the confidence of the estimated steep increases in species richness and abundance is reduced with increasing time since logging. This can be seen particularly well for abundance in Fig. 4b as an increase in the interquartile range enveloping the median estimate for the retention aspens. Nevertheless, it is likely that bryophyte species richness and abundance on retention aspens will approach those of conservation aspens a few decades after logging.

Retention aspens provide high-quality substrate that would be absent from a clear-cut forest. Although the establishing new trees may include some aspens, they will be of very low quality during the first 30 years because they will be small: In the boreal forest a 30-year-old aspen has a diameter of approximately 15 cm [44]. In the clear-cutting forestry system they would be logged by the time they are 80 years old, i.e. most of them would never reach a diameter of more than 40 cm [44]. Therefore they would not be able to support the most demanding species and would be poor habitats for almost all the species in our study (see Fig 2 for species reactions to aspen diameter).

Thus, even though retention trees may not function as effective lifeboats for all bryophytes, they are still likely to meet the second objective of tree retention, i.e. enriching re-established forest stands with structural features that may function as suitable habitats for many species (following [6]). It seems likely that bryophytes can re-colonise retention trees after the surrounding habitat has become suitable again. The re-colonisation is likely to be the combined result of the retention trees growing older and larger and of the re-establishing forest starting to provide more shade, humidity and protection from wind. The high re-colonisation success may be dependent on the fact that most of our retention sites were located close to old-growth forests where the species could disperse from. However, our chronosequence approach leaves uncertainty about the amount of successful colonisations and the source of the dispersal propagules. The predictive model of bryophyte community changes should be verified by further observations and long-term follow-up studies of same retention trees.

All epiphytic species that benefit from lifeboating benefit also from the structural enrichment of the stand because epiphytes require the retained structures as substrates. Two kinds of species may benefit from the additional value of structural enrichment: disturbance-phase species that are able to colonise the retention stand after logging and forest species that are able to re-colonise the re-established stand [12]. Several disturbance-phase lichen species have been found to increase on retention aspens after logging [45], but our results suggest that the number of disturbance-phase epiphytic bryophytes is low. Only two cushion-forming mosses were clearly disturbance-favouring and one of them, *Pohlia nutans*, is commonly found on clear-cuts on several substrates. The other, *Orthotrichum obtusifolium*, is an obligate epiphyte that has earlier been described to occur commonly in intact forests [46], although in some cases its occurrence probability has been found to be positively affected by decreasing shade [47]. Out of the forest species 16 (27% of all species) showed strong positive responses to increasing time since logging, indicating increasingly successful colonisation of the retention aspens with the re-establishment of the surrounding forest. Most of the re-colonising species form mats, including the moss *Sanionia uncinata* and the liverworts *Radula complanata* and *Ptilidium pulcherrimum*, but among the re-colonizing species is also the moss *Dicranum montanum* that grows as short turfs.

### Can retention aspens substitute conservation aspens?

Despite retention aspens being beneficial for the majority of the species, 12 species (20% of all species) were not able to utilize retention aspens as lifeboats and were unable to re-colonise the retention aspens during the few decades after logging. For them intact forests are needed to support long-term persistence of their populations. Most of these species were generally rare in our dataset, including the fan-forming moss *Neckera pennata*. The weft-forming, occasionally epiphytic moss *Hylocomium splendens* is a common forest-floor species with decreasing growth rates in dry and sunny conditions [48], [49]. It declined after logging and was not estimated to recover to its original level during the 30 years, indicating slow recovery of microclimatic conditions and/or slow colonization of the species. Wefts are generally efficient in resource foraging and competition, but their survival is poor in very dry or sunny conditions [19].

When we compared the estimated development of community similarity of both retention and conservation aspens to the modelled old-growth reference community, we observed that soon the trajectory of the community similarity on the retention aspens deviated from that on the conservation aspens (Fig 4c). While this happened, species richness and abundance recovered to a very similar level with the ones in conservation aspens. This is in line with earlier reports showing that species richness is an emergent property of ecosystems and it is maintained on a similar level if resource availability stays on the same level and local compensatory colonisations are possible. On the contrary, community composition is generally much more vulnerable to environmental changes [50]. This observation suggests that although some species are able to lifeboat on the retention aspens and others are able to re-colonize the retention aspens, the overall community structure of the retention aspens is nevertheless likely to remain dissimilar to the conservation aspens. Therefore, it must be concluded that although the retention approach is clearly better than clear-cutting, the retention sites alone are unable to maintain all of bryophyte biodiversity and ensure the long-term persistence of populations.

### Conclusions

Retention forestry has been proposed as one of the most promising solutions to fight against the current rapid loss of forest biodiversity [7], [10]. Our results show that a large proportion of bryophyte species are able to utilize retention aspens as lifeboats or they are able to re-colonise the retention aspens later on and therefore green-tree retention does indeed seem to be an approach that promotes the ecological sustainability of forestry. However, at the same time our results suggest that the responses to logging and the re-colonisation ability are species-specific and it is likely that several species are not able to form viable populations on the retention aspens. Thus, it is clear that the retention approach is not enough on its own but it needs to be accompanied with conservation areas that support those species that are more demanding in terms of their habitat. In addition, as several species may decline on retention aspens after logging but then re-colonise them after a few decades, adjacent old-growth forests with large aspens are needed as potential colonisation sources.

## Supporting Information

File S1 **Site information and original data.**
(XLSX)

File S2 **Details on the statistical analyses.**
(PDF)

File S3 **Species-specific regression coefficients.**
(XLSX)
