# Supplementary material for: Bryophyte Species Richness on Retention Aspens Recovers in Time but Community Structure Does Not
Source: PLoS One. 2014 Apr 7;9(4):e93786. doi: 10.1371/journal.pone.0093786 (PMC3978069; doi:10.1371/journal.pone.0093786)
Supplement: File S2 — Details on the statistical analyses. (PDF) [file pone.0093786.s002.pdf]

Details on the statistical analysis

We analysed the data using the hierarchical community approach of Ovaskainen and Soininen (2011). We denote the species by the index *i*, the tree by the index *j*, and the site of the tree by *k*(*j*). We consider two separate models which share the same structure, but in one model the response variable is presence-absence, whereas in the other it is abundance conditional on presence. In the presence-absence model, we denote by $y_{ij}=1$ if species i was found from tree *j* and by $y_{ij}=0$ if this was not the case. In the abundance model, y_{ij} denotes the abundance of species i on tree j (unit cm^2). In the presence-absence model we applied logistic regression,

$$\mathrm{logit}\left(P\left(y_{ij}=1\right)\right)=L_{ij},$$

whereas in the abundance model we applied linear regression for log-transformed data,

$$\log\left(y_{ij}\right)=L_{ij}+\varepsilon_{ij}.$$

The linear predictor L_{ij} was modelled as

$$L_{ij}=\sum_{c=1}^{l}x_{jc}\beta_{ic}+s_{ik(j)}$$

Here x_{jc} represents the covariate number *c* measured for tree *j* (l=5 is the number of different covariates, see below), β_{ic} is the regression coefficient measuring the influence of covariate *c* on species *i*, and $s_{ik(j)}$ is a site-level random effect on species *i*. The residual ε_{ij} is included only in the abundance model, as in a logistic regression it is not identifiable. The covariate $x_{j1}=1$ models the intercept, x_{j2} is the log-transformed diameter of the tree, x_{j3} is an indicator variable separating retention aspens (x_{j3}=-1) from conservation aspens (x_{j3}=1), x_{j4} is the time since logging (unit year, relevant only for retention aspens), and x_{j5} is the stand age (unit year, relevant only for conservation aspens). The covariates c=2,3,4,5 were normalised to zero mean and unit variance to make their effect sizes measured by the regression coefficients comparable with each other.

The site-level random effects ($s_{ik(j)}$) were assumed to be distributed according to the multivariate normal distribution with covariance structure

$$\mathrm{Cov}[s_{ik},s_{i'k'}]=\delta_{kk'}\sigma_S^2(\delta_{ii'}+\rho(1-\delta_{ii'})),$$

where $\delta_{kk'}$ denotes Kronecker delta with $\delta_{kk'}$=1 if $k=k'$ and $\delta_{kk'}$=0 if this is not the case, i.e. the effect was assumed to be independent between the sites. Here σ_s^2 models the amount of such variation among sites that is not captured by the site-level covariates (c=3,4,5), and ρ is the level of correlation among species. If ρ=0, the species respond independently to un-modelled variation among sites, whereas ρ=1 corresponds to variation in species richness but not in species composition.

The residual ε_{ij} in the abundance model was assumed to be normally distributed and have the covariance structure

$$\mathrm{Cov}\left[\varepsilon_{ij},\varepsilon_{i'j'}\right]=\delta_{ii'}\delta_{jj'}\sigma_R^2.$$

The species-specific models were combined by a community-level model as

$$\boldsymbol{\beta}_{i.}\sim N(\boldsymbol{\mu},\boldsymbol{\Sigma}).$$

Here $\boldsymbol{\beta}_{i.}$ is a vector that is formed by the responses (regression coefficients β_{ic}) of the species *i* to the five covariates,

$$\boldsymbol{\beta}_{i.}=\left[\begin{array}{ccccc}\beta_{i1} & \beta_{i2} & \beta_{i3} & \beta_{i4} & \beta_{i5}\end{array}\right]^T$$

$\boldsymbol{\mu}$ is a vector that is formed by the average responses (μ_c) of the species to the five covariates,
$\boldsymbol{\mu} = \left[\mu_1 \; \mu_2 \; \mu_3 \; \mu_4 \; \mu_5 \right]^{\mathrm{T}}$

$\boldsymbol{\Sigma}$ is a variance-covariance matrix that includes variation among species in their responses to the environmental covariates (variances on the diagonal elements) and co-variation among responses to different covariates (covariances on the off-diagonal elements),

$$\boldsymbol{\Sigma} = \begin{bmatrix} \sigma_1^2 & \sigma_{12} & \sigma_{13} & \sigma_{14} & \sigma_{15} \\ \sigma_{21} & \sigma_2^2 & \sigma_{23} & \sigma_{24} & \sigma_{25} \\ \sigma_{31} & \sigma_{32} & \sigma_3^2 & \sigma_{34} & \sigma_{35} \\ \sigma_{41} & \sigma_{42} & \sigma_{43} & \sigma_4^2 & \sigma_{45} \\ \sigma_{51} & \sigma_{52} & \sigma_{53} & \sigma_{54} & \sigma_5^2 \end{bmatrix}$$

The correlation coefficient between the responses to two covariates, c_1 and c_2, is defined as

$$\rho_{c1,c2} = \Sigma_{c1,c2} / \sqrt{\Sigma_{c1,c1}\Sigma_{c2,c2}}.$$

We fitted the presence-absence model and the abundance model independently of each other. We used Bayesian inference, and thus prior distributions needed to be defined for the community-level parameters $\boldsymbol{\mu},\boldsymbol{\Sigma}$, for the parameters related to the site-level random effects (σ_s^2 and ρ), and for the residual variance parameter (σ_R^2, relevant only for the abundance model). For the ease of posterior sampling, we assumed the conjugate normal-inverse-Wishart prior (Gelman et al. 2004) for ($\boldsymbol{\mu},\boldsymbol{\Sigma}$), i.e. $\boldsymbol{\Sigma}$~Inv–Wishart$_{v_0}(\boldsymbol{\Lambda}_0^{-1})$ and $\boldsymbol{\mu}|\boldsymbol{\Sigma} \sim N(\boldsymbol{\mu}_0,\boldsymbol{\Sigma}/\kappa_0)$. We set $\boldsymbol{\Lambda}_0$ to identity matrix, $\boldsymbol{\mu}_0$ to zero vector, $\kappa_0=0$ and $v_0=c$. For ρ we assumed a uniform prior in (0,1) and for $\log(\sigma_s^2)$ and $\log(\sigma_R^2)$ we assumed a normal prior with mean -1 and variance 1.

We fitted the models to data using a slightly adapted version of the MCMC scheme of Ovaskainen and Soininen (2011). The proposal distributions needed in the Metropolis-Hastings algorithm were adjusted adaptively during the burn-in (with 10,000 iterations) to obtain an optimal acceptance ratio, after which the posterior was sampled with 50,000 iterations thinned to 1,000 samples. The estimation was performed with Mathematica 7.0.

Details on the scenario comparisons

We used the fitted models to compare the development of bryophyte communities between retention and conservation aspens. We considered as the starting point an aspen tree with 30 cm diameter located in a forest with a stand age of 80 years. We then assumed that the forest was logged (in which case the aspen became a retention tree) or conserved, and examined how the community on the aspen would evolve over time until 30 years since logging (for the retention aspen), or until the stand age reached 150 years (for the conservation aspen). We assumed that the diameter of the aspen grew linearly so that it reached 60 cm for the stand age of 150 years. For these scenarios, we predicted the expected species richness (based on the presence-absence model) and the abundance (cm^2) of all bryophytes (based on probability of presence from the presence-absence model multiplied by abundance conditional on presence from the abundance model). We also predicted how similar the community structure (predicted by the presence-absence model) would be to a reference community of an old-

growth aspen, defined here as the modelled community of an aspen that has 60 cm diameter and occurs on a conservation site with stand age of 150 years. We followed (Ovaskainen and Soininen, 2011) by measuring community similarity between the reference (R) and focal (F) communities through the correlation

$$\frac{\mathrm{Cov_{RF}}}{\sqrt{\mathrm{Var_R} \times \mathrm{Var_F}}}.$$

The variances and covariances were computed as

$$\mathrm{Cov}_{RF} = \mathbf{x}_R^T \mathbf{\Sigma} \mathbf{x}_F + \delta_{k(R)k(F)} \sigma_S^2,$$

where $\mathbf{x}_R$ and $\mathbf{x}_F$ are the vectors of environmental covariates for the reference and focal communities, respectively, and the superscript T denotes the transpose of a matrix.
